# Rapid processing of fearful faces relies on the right amygdala: evidence from individuals undergoing unilateral temporal lobectomy

**DOI:** 10.1038/s41598-020-80054-1

**Published:** 2021-01-11

**Authors:** David Framorando, Eleanor Moses, Lore Legrand, Margitta Seeck, Alan J. Pegna

**Affiliations:** 1 School of Psychology, The University of Queensland, Saint Lucia, Brisbane, QLD 4068 Australia; 2 Unit for Presurgical Evaluation of Epilepsy, Neurology Clinic, Geneva University Hospitals, 1205 Geneva, Switzerland

**Keywords:** Cognitive neuroscience, Amygdala, Visual system

## Abstract

Facial expressions of emotions have been shown to modulate early ERP components, in particular the N170. The underlying anatomical structure producing these early effects are unclear. In this study, we examined the N170 enhancement for fearful expressions in healthy controls as well as epileptic patients after unilateral left or right amygdala resection. We observed a greater N170 for fearful faces in healthy participants as well as in individuals with left amygdala resections. By contrast, the effect was not observed in patients who had undergone surgery in which the right amygdala had been removed. This result demonstrates that the amygdala produces an early brain response to fearful faces. This early response relies specifically on the right amygdala and occurs at around 170 ms. It is likely that such increases are due to a heightened response of the extrastriate cortex that occurs through rapid amygdalofugal projections to the visual areas.

## Introduction

Over the last 2 decades, studies investigating the visual processing of facial expressions of emotions have identified the participation of structures extending beyond the usual visual areas, in particular with the involvement of the amygdala where fearful faces are concerned^1–6^. On the other hand, some studies have pointed out a crucial role of the right amygdala in emotion processing^2,4,7–9^. Furthermore, it has been shown that the amygdala is activated when faces are presented subliminally to prevent conscious detection. More strikingly, even after the destruction of the primary visual cortex and the appearance of cortical blindness, amygdala activation remains present following visual presentations of facial expressions^9^. Although several visual pathways have been suggested to account for such effects^10^, one influential view has postulated that the amygdala might receive information regarding facial expressions through a direct, subcortical route, which bypasses the primary visual cortex. This pathway would allow unconscious processing to occur even when the main thalamo-striate route to the cortex is damaged or insufficiently activated^9,11–14^.

Evidence has suggested that this subcortical route engages projections from the retina to the superior colliculus, the pulvinar and the amygdala^15^. It is thought that this phylogenetically older pathway may have survived as an alternative route due to its evolutionary usefulness^16–18^. Indeed, if rapid avoidance is to occur for threatening stimuli, a faster, albeit crude, pathway may allow precious milliseconds to be gained for the organism’s response. Hence, the existence of the subcortical pathway may reside in its rapid responsiveness to emotional or relevant stimuli. In the healthy brain, it has been hypothesized that this rapid activation of the amygdala may enhance the visual cortical response to relevant stimuli through retrograde feedback projections to extrastriate regions^6^. If the cortical route is damaged, this subcortical pathway would lead to affective blindsight in patients with cortical blindness, and unconscious processing in subliminal procedures^11,12,19–21^.

One outstanding question regarding this hotly debated subcortical pathway is whether or not it is truly rapid. To answer this question, an appropriate experimental investigation of this question must involve a method that uses a high temporal resolution.

A number of event-related potential (ERP) studies have examined the timing of cortical activation in response to emotional faces and have observed emotion-based modulations of the early components. Both the P1^22,23^ and N170 components^21,24–27^ are reportedly enhanced by faces expressing fear and by auditory signals of distress^28^. Importantly, a more recent review^29^ suggested a more robust effect for the latter component, while P1 and P2 modulations appear to be highly variable and inconsistent. More compellingly, subliminal presentations of fearful faces also appear to produce an enhanced early fronto-central activity^19^ or a N170 response^21^, and lateral subliminal presentations in dot-probe tasks produce lateralised N170 effects^27^. These observations, coupled with the observations from fMRI and PET^30,31^ showing that the amygdala is activated by subliminal faces, suggest that the early modulation could be driven by the amygdala and would therefore support that its activation occurs rapidly enough for it to enhance extrastriate activity as suggested by Vuilleumier et al.^6^.

In an attempt to confirm this, Rothstein et al.^5^ compared the ERP response to fearful and neutral faces in a small group of epileptic patients. The findings indicated an enhancement of the P1 for fearful compared to neutral faces that was correlated with the degree of amygdala atrophy, again suggesting that the early ERP modulation was dependent on the integrity of the amygdala.

The current investigation therefore aimed to determine the timing of amygdala involvement in the visual processing of emotional (fearful) facial expressions by examining ERP responses in patients following right or left anterior temporal lobectomy (ATL) that included the amygdala. We recorded EEG in patients with left and right ATL and a group of healthy controls who were presented with fearful, happy and neutral faces, as well as a control category (vegetables; 300 ms) during a 1-back task. The faces were presented at the centre of the screen for 300 ms and were followed by a blank (black) screen that appeared for 1200 ms. ERP components (P1, N170 and P2) to the three facial expressions and vegetables were compared in the 3 groups. We hypothesised that controls would show an enhanced N170 due to feedback projections from amygdala, while the absence of an amygdala in ATL patients would disrupt this effect.

## Results

A sensitivity analysis (α err prob = 0.05; total sample sizes: 16 (control group) vs. 8 (patients group); number of groups: 1(control group) vs. 2 (patients group); number of measurements: 6; correlations among rep measures: 0.8; sphericity: 0.7) showed that the present study is powered to detect effect sizes <  = of 0.30.

An analysis of covariance was carried out on the P1, N170, and P2 amplitudes using Stimulus Category (Fear, Happy, Neutral, Vegetable), Electrode Hemisphere (Left vs. Right) and Participant Group (Left ATL, Right ATL, Control) as factors, and age as a covariate. The covariate age was included to determine if age had a differential effect on amplitude, as this varied somewhat across participant groups.

*P1.* The 4 (Fear, Happy, Neutral, Vegetable) × 2 (Left, Right Hemisphere) × 3 (Left ATL, Right ATL, Control) ANCOVA revealed a significant main effect of Hemisphere, *F*(1, 23) = 6.89, *p* = 0.015, η2 = 0.23. The P1 deflection was stronger over the right (4.39 µV ± 0.65) compared to the left (4.03 µV ± 0.47) hemisphere. No other effects were significant.

*N170.* The 4 × 2 × 3 ANCOVA revealed a main effect of Stimulus Category *F*(3, 69) = 19.30, *p* < 0.001, η2 = 0.46, a main effect of Hemisphere, *F*(1, 23) = 6.95, *p* = 0.015, η2 = 0.23, as well as a Hemisphere x Group interaction, *F*(2, 23) = 4.71, *p* = 0.019, η2 = 0.29, and an Emotion x Group interaction, *F*(6, 69) = 12.29, *p* < 0.001, η2 = 0.52. The N170 deflection was stronger over the right (− 5.84 µV ± 0.77) than over the left (− 5.24 µV ± 0.58) hemisphere. All faces produced a larger N170 deflection than vegetables (− 1.67 µV ± 0.53), *F*(1, 23) = 76.41, *p* < 0.001, η2 = 0.77. Moreover, fearful faces (− 7.43 µV ± 0.80) produced a larger N170 than happy (− 6.68 µV ± 0.75) and neutral faces (− 6.37 µV ± 0.68), *Fs*(1, 23) > 15.79, *ps* < 0.001, η2 > 0.41, whereas happy faces did not significantly differ from neutral faces (*p* = 0.374). In addition, we ran focused-cell contrasts between emotions across the three groups. Faces produced a greater N170 deflection than vegetables (means shown in Table 1) in Left and Right ATL patient groups, *Fs*(1, 23) > 4.89, *ps* < 0.038, η2 > 0.017. In the control group, fearful and happy faces significantly differed from vegetables, whereas neutral faces fell short of the threshold for significance (*p* = 0.057).

**Table 1:** Mean amplitude values and standard errors of the N170 for fearful, happy, neutral faces and vegetables for the left and right ATL patients and for the control group.

	Left ATL	Right ATL	Control
Fearful Faces	(− 12.41 µV ± 1.67)	(− 7.25 µV ± 1.52)	(− 2.63 µV ± 0.93)
Happy Faces	(− 10.56 µV ± 1.53)	(− 7.07 µV ± 1.40)	(− 2.42 µV ± 0.85)
Neutral Faces	(− 9.30 µV ± 1.39)	(− 7.64 µV ± 1.27)	(− 2.18 µV ± 0.77)
Vegetables	(− 2.68 µV ± 1.08)	(− 2.12 µV ± 0.99)	(− 0.21 µV ± 0.61)

*P2.* The 4 × 2 × 3 ANCOVA revealed a main effect of Stimulus Category *F*(3, 69) = 8.77, *p* < 0.001, η2 = 0.28 and a Stimulus Category x Group interaction, *F*(6, 69) = 20.13, *p* < 0.001, η2 = 0.64. Focused-cell contrasts revealed that vegetables (7.73 µV ± 0.67) produced a stronger P2 compared to fearful (2.47 µV ± 0.65), neutral (2.93 µV ± 0.59) and happy (2.92 µV ± 0.73) faces, *Fs*(6, 69) > 43.28, *ps* < 0.001, η2 > 0.65. Fearful faces did not significantly differ from neutral and happy ones. In addition, we ran focused-cell contrasts between emotions across the three groups. Faces differed from the vegetables in the Left and Right ATL groups, *Fs*(1, 23) > 11.53, *ps* = 0.002, η2 > 0.33, whereas no significant differences was found for the control group (*ps* > 0.441).

**Figure 1:**
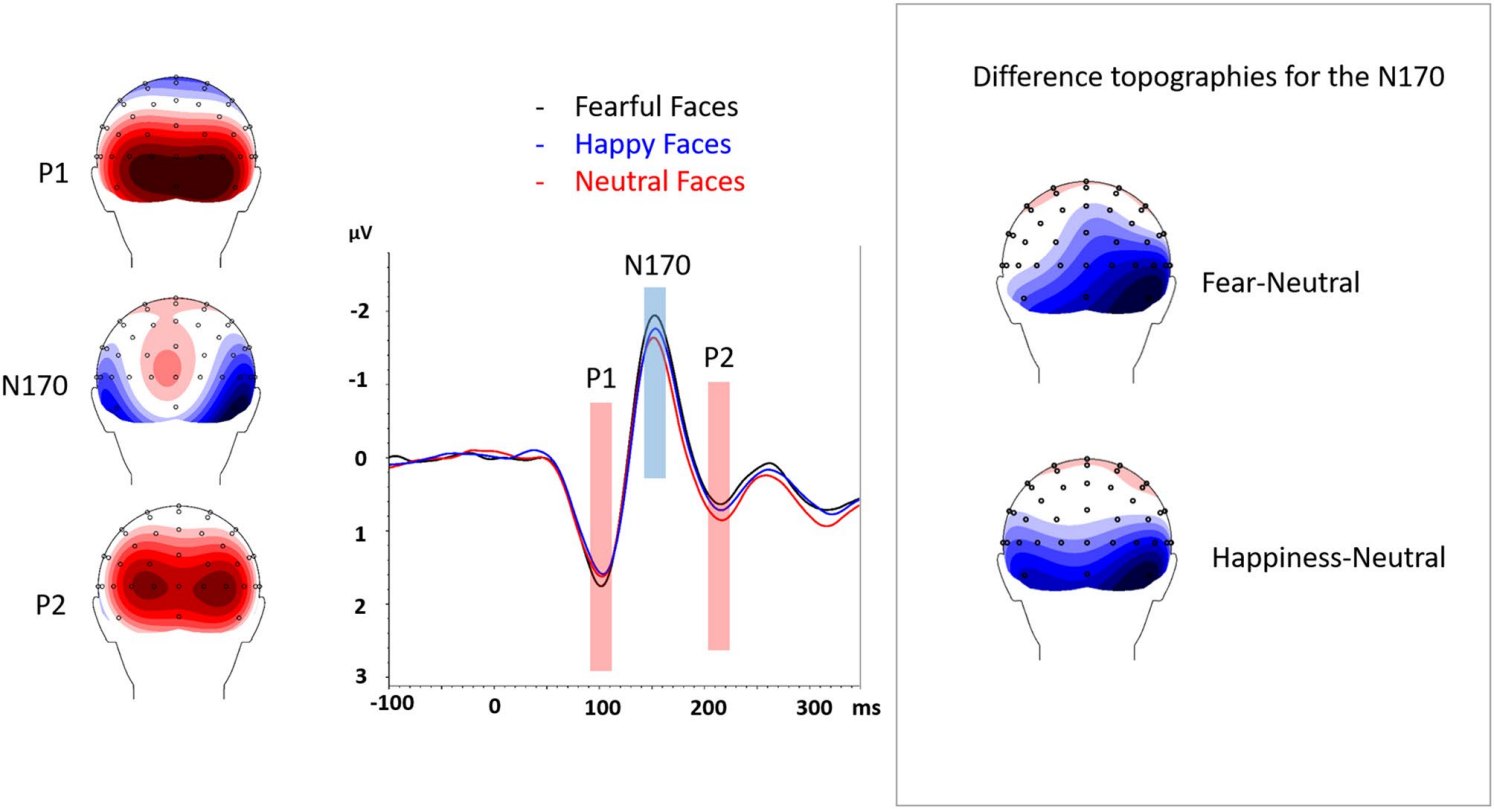
The N170 is shown for fearful faces (black) and neutral faces (red) conditions. Left and right leads are collapsed. On the right, difference topographies are shown for fearful minus neutral conditions and for happy minus neutral conditions at the N170.

In order to test specifically our main hypothesis (i.e., effects of lobectomy on the N170 response to fearful facial expressions), we further carried out two separate repeated-measures ANOVAs on the N170 amplitudes in the control and patient groups. The two groups were examined separately, comparing neutral, happy and fearful expressions. As age did not interact with emotion in either group, this factor was not included in the follow-up analysis (two separate ANCOVAs revealed that age was not related to Emotion in the patient and in the control group, *ps* > 0.426) (Fig. 1).

### Control group

**Figure 2:**
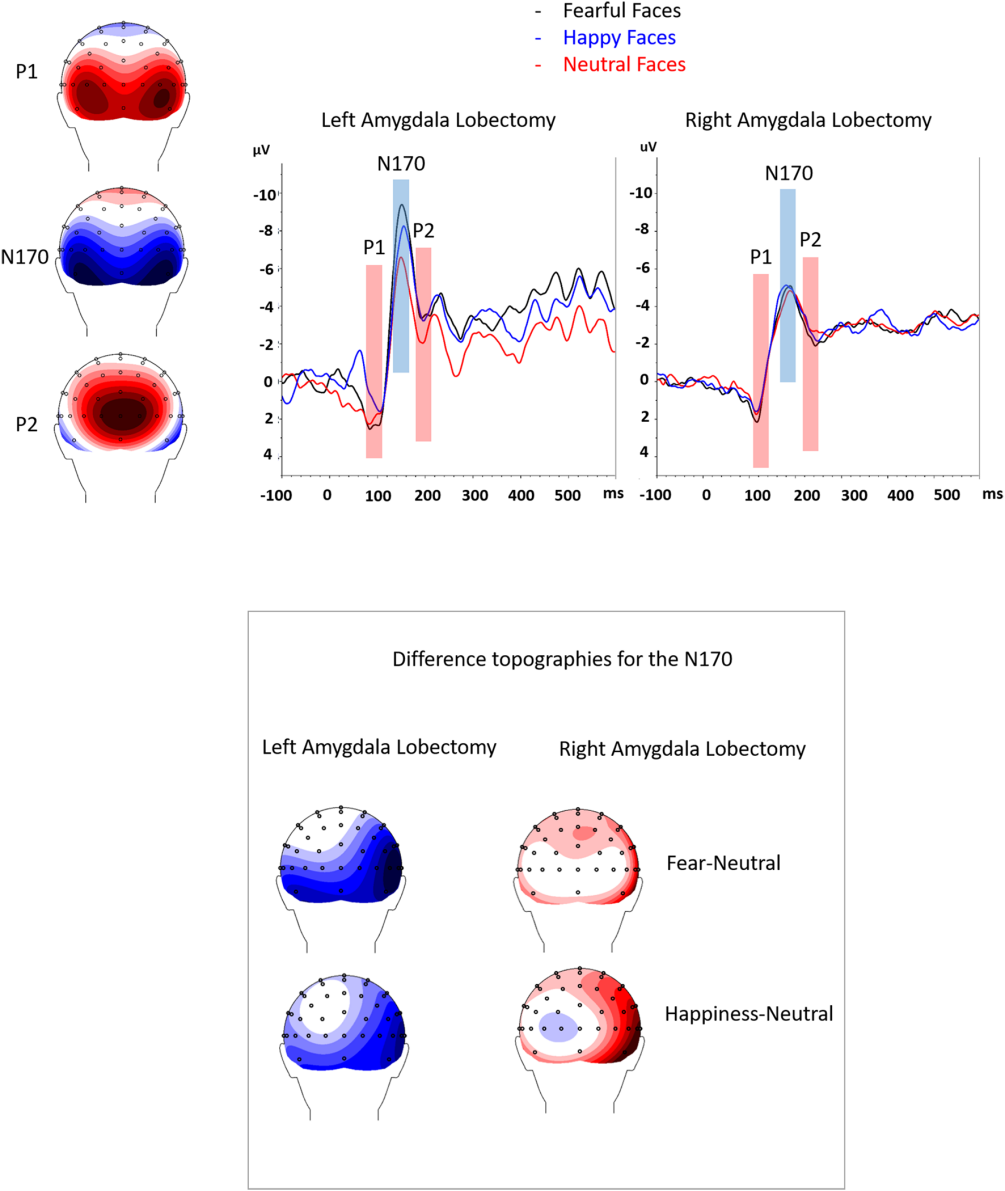
The N170 for the left and right amygdala lobectomy is shown for fearful faces (black), neutral faces (red) and happy faces (blue) conditions. Left and right hemispheres are collapsed. On the bottom, we displayed the difference topographies between the fearful and the neutral conditions and between the happiness and the neutral conditions for the N1 of patients with left and right amygdala lobectomy.

*N170.* The 3 (Fear, Happy, Neutral) × 2 (Left, Right Hemisphere) ANOVA revealed a main effect of Hemisphere, *F*(1,15) = 12.32, *p* = 0.003, η2 = 0.45, and of Emotion, *F*(2,30) = 4.41, *p* = 0.034, η2 = 0.23. The N170 deflection was greater in the right (-2.79 µV ± 0.44) compared to the left (-2.03 µV ± 0.34) channels. As illustrated in Fig. 2, focused-cell contrasts revealed that fearful faces (-2.63 µV ± 0.38) produced a stronger N170 deflection compared to neutral (-2.18 µV ± 0.38) and happy (-2.42 µV ± 0.40) faces, *Fs*(1, 15) > 3.26, *ps* < 0.045, η2 > 0.17 (one-tailed), whereas happy faces did not significantly differ from neutral faces (*p* = 0.103). The Emotion x Hemisphere interaction was not significant, *F*(2, 30) > 3.04, *ps* = 0.06, η2 > 0.17.

### Patients with amygdala lobectomy

*N170.* Results revealed a significant Emotion x Amygdala Lobectomy interaction, *F*(2, 18) = 16.73, *p* = 0.022, η2 = 0.40. To decompose this interaction, focused-cell contrasts were run separately for left and right lobectomized patients. As seen in Fig. 2, in patients with left amygdala lobectomy, fearful faces (− 12.41 µV ± 2.58) produced a stronger N170 deflection relative to neutral (− 9.30 µV ± 2.09), and happy (− 10.56 µV ± 2.33) faces, *Fs*(1, 9) > 14.62, *ps* < 0.004, η2 > 0.62, while happy faces did not differ significantly from neutral faces (*p* = 0.199). By contrast, no significant effect emerged in patients with right amygdala lobectomy (*ps* >  = 0.511). No other effects were significant (*ps* > 0.062).

## Discussion

The results of this study reveal that, in healthy controls, fearful faces produce an enhanced N170 component compared to neutral faces, corroborating a number of previous reports^21,24–27,32^. More importantly, this enhancement continued to be observed in a group of patients who had undergone left temporal resection, but not those whose resection had been on the right, suggesting that the N170 enhancement requires a functional right amygdala.

The idea that early ERP components are enhanced for emotional expressions emerged almost two decades ago. For example, Batty and Taylor^24^ reported that fearful faces produced a greater N170 than neutral and happy faces. Similarly, modulations of the N170 for fearful faces were subsequently reported in tasks where the facial stimuli were irrelevant^25^, or when they were presented subliminally^21^. Similar findings seem to apply to other emotional expressions such as crying faces^26^, and even to fearful body expressions^32^. Other authors suggested an even earlier modulation, reporting P1 enhancements for fearful faces. For example, Batty & Taylor^24^ observed a P1 increase for fearful faces, a finding echoed by others^23^. Moreover, Eimer and Holmes^22^ also reported effects in the 100 ms range, but over anterior electrodes, an effect that the authors attributed to enhanced attentional processes. The reasons for which this early ERP enhancement occurs has remained a point of discussion so far. One point of view that has been discussed at length is that emotional faces might differ in their low-level features, which could produce an early modulation of the ERP response. For example, fearful expressions are generally characterised by widely opened eyes, thereby increasing the brightness in the eye region. This could naturally lead to an increase in the early sensory responses that are sensitive to brightness. With this in mind, one experiment inverted the faces to disrupt emotion recognition while maintaining the low-level features^22^. The effect of this manipulation was to attenuate the ERP differences produced by the emotional expressions. Had the effects been driven by low-level features rather than emotion, such a manipulation would not have been found. Another approach consists in ensuring that no systematic difference exists in the brightness of the emotional stimuli. In doing so, the likelihood of this factor driving the effect is reduced. Along these lines, differential effects between neutral and fearful faces were still reported when stimuli were controlled in brightness and contrast using a histogram-based method for equalizing perceptual variations^25^. More recently, a study examined the ERP response to emotional faces and their scrambled Fourier transformed version, in which low-level visual features were preserved^33^. The findings confirmed that emotional expressions modulated both the P1 and N170, however, the P1 response was modulated by both scrambled and intact versions, while only the N170 was modulated by emotional expression. This suggests that the P1 modulations are due to low-level differences across stimuli, while the N170 is specifically tied to emotions.

Our current study circumvents any bias due to low-level characteristics since the same stimuli were used for the comparison across groups of participants (i.e., any difference in luminance between categories is the same across groups). Hence, the loss of an N170 modulation by the right-lobectomy group alone demonstrates an emotion-dependent effect of the right amygdala and precludes a low-level explanation for this modulation. This raises the question about the mechanism underlying the enhancement of the N170 by fearful faces.

Several authors have proposed that emotional/threatening stimuli activate a subcortical pathway that in turn increase the neuronal activity in visual areas through feedback from the amygdala^6,34^. More specifically, emotional/threatening stimuli have been posited to activate a rapid subcortical pathway to the amygdala, allowing coarse but rapid processing of relevant stimuli^18,35^. Ample evidence has been provided to corroborate the existence of this extra-geniculate visual pathway^4,18,36^, allowing the amygdala to be activated even in the absence of a primary visual cortex^9^. One role of this parallel pathway is thought to be the enhancement of visual processing for relevant stimuli, such as fearful faces. Addressing this question, Vuilleumier et al.^6^ compared the BOLD response in extrastriate visual areas to fearful and neutral faces in patients with temporal lobe epilepsy, and patients who presented with hippocampal sclerosis which included or excluded the amygdala. The visual extrastriate response was found to be enhanced for fearful faces compared to neutral expressions in all patients except those with amygdalar sclerosis. This led to the conclusion that the amygdalae were responsible for the increase in visual extrastriate activity for emotional faces, suggesting feedback activation from this structure to visual areas. However, to confirm whether a causal effect is present, the temporal dynamics of neural activation must be examined. A subsequent study by Rotshtein et al.^5^ sought to identify the periods during which the amygdala affected the electrical response of the brain. Their study again examined epileptic patients who presented hippocampal and amygdalar sclerosis, or hippocampal sclerosis alone, this time using an ERP paradigm with fearful and neutral faces. Their results revealed that the presence of amygdala sclerosis dampened the P1 enhancement observed for fearful faces and that this amplitude was correlated with the degree of atrophy. These findings confirmed an early modulation of the ERP response although the involvement of anterior channels is inconsistent with the hypothesis of extrastriate activity. On the other hand, our findings identify the N170 as the component of interest, revealing changes over expected channels and consistent with extrastriate activity (although no source localisation was performed). Moreover, our current findings were obtained from patients following amygdala resection, showing that the effect was highly lateralized. Specifically, participants with right amygdala damage showed no modulation of the N170. By contrast, participants who presented damage to the left, but not the right amygdala showed that fearful faces produced a stronger N170 compared to happy and neutral faces.

Our current finding is the first combined amygdalectomy/ERP study to reveal an asymmetry of function of the amygdala for emotion processing. A small number of experiments have previously reported asymmetries following the presentation of fearful stimuli^1,37,38^. For instance, one experiment reported that passively watching fearful faces activated the left amygdala more than watching neutral faces^38^. Another experiment, reported that the left amygdala responded more to fearful faces in a dot-probe task^37^. In this study, a fearful face could appear simultaneously with a neutral face either in the left or the right visual field. In the control condition, two neutral faces were presented. Results revealed a heightened left amygdala activation when fearful faces were present in the left visual field compared to the control condition. The authors therefore proposed that the left amygdala mediates attentional effects to masked fearful faces. Contradicting these observations however, other papers have highlighted the role of the right amygdala in emotion processing^2–4,7–9,30,31^. For example, Cecere et al.^2^ reported that consciously perceived happy faces elicited an increased N170 when an unseen fearful face appeared simultaneously in the blind field of a right hemianopic patient. The authors concluded that implicit subcortical processing of fearful signals could influence face encoding only when the right hemisphere was intact. Elsewhere, Morris et al.^4^ reported that unseen fearful faces and fear-conditioned faces activated the right but not left amygdala in control participants. These authors concluded to the existence of a subcortical pathway to the right amygdala that processes behaviorally relevant visual events independently of the extratriate route.

The results of our current study corroborate the latter studies, emphasizing the right amygdala’s essential role for emotional processing. In line with previous suggestions^6^, we hypothesize that this may be rendered possible by rapid subcortical projections to the amygdala that precede the feedforward sweep of information through the geniculate pathway. Indeed, the amygdala has been posited to project to the primary virtual cortex, and thus can modulate the processing of visual information in this area^34,39^. By triggering this fast-track pathway, fearful faces would activate amygdala, leading to an increase in the extrastriate response. By contrast with right amygdalectomies, patients with left amygdala removal likely retain the use of the extra-geniculate pathway for fearful face processing. This in turn could lead to an enhanced extrastriate response and enhanced stimulus encoding^2^, thereby giving rise to N170 modulations^40^.

Taken together, our results demonstrate that the early ERP modulation—in this case the N170—for fearful faces necessitates the integrity of the amygdala, and more importantly that this N170 enhancement relies on the right amygdala. It is likely that projections to the right amygdala provide a route for the processing of behaviorally relevant stimuli such as fearful faces, which lead to a heightened response of the extrastriate cortex via rapid amygdalofugal projections to the visual areas.

## Methods

### Participants

The study was approved by The Human Research Ethics Committee at The University of Queensland. All methods were performed in accordance with the guidelines and regulation at University of Queensland.

#### Control participants

Sixteen healthy participants (3 males) took part in the study. Participants were recruited through the University of Queensland research participation program from a first year psychology course, and were awarded partial course credit for their participation. Mean age was 24.10 years (± 8.34). All had normal or corrected-to-normal vision and had no self-reported psychiatric or neurological condition.

#### Amygdalectomy patients

Fourteen patients (8 female) with pharmaco-resistant epileptic seizures who underwent amygdalohippocampectomy of the left (8 individuals) or right (6 individuals) were initially recruited from the Unit for Presurgical Evaluation of Epilepsy (Neurology Clinic, Geneva University Hospital). The data of two patients was discarded for the study due to a high number of EEG artefacts such as for example eyeblinks on the stimulus. The average age of the resulting group of participants was 37 years; epilepsy onset varied between 1 year of age and 27 years of age. The study was performed between 3 months and 17 years after surgery. Patient details are provided in Table 2 in method section. They suffered from intractable epilepsy between 6 and 54 years until surgical alleviation of the disease. Participants had normal or corrected-to-normal vision.

**Table 2:** Summary of patient group. Age is in years at the time of the experiment.

Patient	Gender	Age	Surgery laterality	Temporal pole excision	Age of seizure onset	Years since surgery	Seizure Type	seizure free
1	M	28	L	No	7	14	PC	Yes
2	F	42	L	No	12	6	PCG	Yes
3	F	26	L	Yes	1	14	P	Yes
4	F	28	L	Yes	n/a	2	PCG	Yes
5	M	49	L	Yes	15	14	PC	Yes
6	F	47	L	Yes	12	8	PCG	Yes
7	F	22	R	Yes	7	3	PC	Yes
8	F	56	R	Yes	2	.25	PG	No
9	M	35	R	Yes	11	10	PG	Yes
10	F	46	R	Yes	14	17	PC	Yes
11	F	31	R	Yes	27	1	PC	Yes
12	M	42	R	No	12	15	PCG	Yes

All patients underwent unilateral removal of the amygdala and hippocampus either on the left (L) or on the right (R). Patients whose surgery included the anterior temporal pole are indicated as “yes’ or “no” in the 5th column. Type of seizures are indicated as P = partial simple, PC = partial complex, PCG = partial complex secondarily generalized. n/a = not available.

### Stimuli

A total of 30 greyscale photographs (236 × 236 pixels) faces expressing fearful, happy or neutral expressions (10 per category, 5 males) were selected from the K-DEF database^41^. The faces were cropped at the hairline to show only facial features. Faces were presented on a black background (RGB: 0, 0, 0). The program ImageJ (http://rsbweb.nih.gov/ij/index.html) was used to calculate the luminance across categories. All picture modifications were made with Adobe Photoshop CS3. Faces stimuli were equated for luminance and were presented in the centre of the monitor (21″ Hewlett Packard, LCD screen) at a refresh rate of 60 Hz, situated at 115 cm from the subject with minimal lighting.

Greyscale photographs of 20 common vegetables (onions, carrots, aubergines, etc.) were added as distracters. These stimuli were adjusted to the same size and brightness levels as the face stimuli.

### Procedure

A one-back procedure was used. The photographs were presented for 300 ms in the centre of the screen. On 10% of the trials, the stimulus would be presented twice in immediate succession. To ensure that attention was maintained on the stimuli, participants were instructed to respond to these immediate repetitions by pressing a key on a standard keyboard. The order of presentation of the stimuli was randomised across participants. Instructions were delivered verbally and were repeated in writing on the screen. Participants were told to maintain their gaze on a central fixation cross. They were informed that photographs of faces and vegetables would be presented and were asked to press the designated key on the keyboard whenever a picture was presented twice in succession.

A white fixation cross was presented on a black background for random durations between 500 and 1000 ms. This was followed by the stimulus that lasted 300 ms. A blank (black) screen then appeared for 1200 ms. At the end of each block, a self-paced break was observed prior to initiating the following block.

For patient recordings, a camera was attached on the experiment presentation screen to allow for constant monitoring via the video as well as the EEG recording in case of any difficulty, or the onset of an epileptic seizure, and the video and EEG were monitored in the experimenter control group during the procedure. The experimental procedure was run using a dedicated software (E-prime v2; www.pstnet.com/eprime).

Three emotional expressions were used (fear, happy and neutral), displayed by 10 different individuals. These 30 photographs were presented randomly for a total of 8 times throughout the experiment, totalling 240 stimuli. An additional 80 photographs representing vegetables were included, and 10% of the stimuli were randomly presented as an immediate repetition for the one back procedure (= 32 samples). Participants thus viewed 352 stimuli in total, presented in 4 blocks of equivalent duration.

The total duration of the procedure (4 blocks + breaks) was approximately 25 min.

The study was approved by the Ethics Committee of Geneva University Hospitals (epileptic patients) and the Ethics Committee of the University of Queensland (healthy controls). Participants gave their written informed consent to participate before beginning the experiment.

#### ERP recording

Continuous EEG was acquired at 1024 Hz using an AD-Box ActiveTwo amplifier (Amsterdam, The Netherlands) and 64 equally-spaced scalp electrodes referenced to Cz. Two external electrodes EOG were placed on the face in order to monitor eye blinks and saccades (one on the outer canthus of the right eye and one above the right eyebrow). A trigger pulse was sent by the stimulus-delivering PC to the EEG-acquisition PC upon appearance of the visual stimulus. The timing of the markers was verified during preparation of the paradigm by comparing stimulus presentation on the screen (using a photodiode) and marker onset on the EEG signal.

EEG signal analysis was performed using BrainVision Analyzer 2.1 (Brain Products, Gilching Germany). All trials in which participants responded were excluded from EEG analysis. Epochs were first established from 100 ms before, to 350 after stimulus onset. Bad electrodes were removed and re-interpolated using 3D splines^42^. ERPs were then baseline corrected using the 100 ms pre-stimulus period. The signal was filtered offline between 0.1 and 30 Hz, with Cz maintained as the reference. We computed the mean amplitude for the P1, N170, and P2 components. Mean amplitudes were established by visually determining the electrodes with the maximum amplitude at the regions of interest (ROIs), as well as the temporal occurrence in the grand means.

#### ERP Processing

Analyses were performed on the mean amplitudes of the P1, N170 and P2 components. All 3 components were measured at their maximal occurrence, determined in time windows at respectively: 95–120 ms, 145–170 ms and 205–230 ms for both the patient group and the control group. The sites were based on electrodes of maximal activity during these intervals and were consistent with those typically used to investigate these components (PO8/PO7, P7/P8, P9/P10 for the P1; P7/P8, P9/P10 and TP9/TP10 for the N170; and O1/O2/Oz/POz for P2).

An analysis of covariance was carried out on the P1, N170, and P2 amplitudes using Stimulus Category (Fear, Happy, Neutral, Vegetable), Electrode Hemisphere (Left vs Right) and Participant Group (Left ATL, Right ATL, Control) as factors, and age as a covariate. Then two separated ANOVAs were performed for the control and the patient groups to test the effects of fearful facial expressions on the N170.

For the control group, the analyses consisted of a 3 (Emotion: neutral, fearful, happy faces) × 2 (Hemisphere: left, right) repeated-measures ANOVA. The analyses of the patient group consisted of a 3 (Emotion: neutral, fearful, happy faces) × 2 (Hemisphere: left, right) repeated-measures ANOVA with left and right ATL included as a categorical factor. Violations of sphericity and p-values were corrected according to the epsilon of Greenhouse–Geisser or Huynh–Feldt.
